# Development of the Vietnamese Healthy Eating Index

**DOI:** 10.1017/jns.2022.44

**Published:** 2022-06-09

**Authors:** Duong T. T. Van, Laura Trijsburg, Ha T. P. Do, Kayo Kurotani, Edith J. M. Feskens, Elise F. Talsma

**Affiliations:** 1 Division of Human Nutrition and Health, Wageningen University and Research, Wageningen, The Netherlands; 2 Department of Nutrition and Food Science, University of Medicine and Pharmacy at Ho Chi Minh City, Ho Chi Minh City, Vietnam; 3 National Institute of Nutrition, Ministry of Health, Hanoi, Vietnam; 4 Department of Health Science, Showa Women's University, Tokyo, Japan; 5 Department of Nutritional Epidemiology and Shokuiku, National Institutes of Biomedical Innovation, Health and Nutrition, Tokyo, Japan

**Keywords:** Dietary quality, Food-based dietary guidelines, Healthy Eating Index, Vietnamese adults, A4NH, The CGIAR Research Program on Agriculture for Nutrition and Health, AFE, Adult Female Equivalent, AME, Adult Male Equivalent, *E* %, energy percentage, FBDG, food-based dietary guidelines, FCT, Food Composition Table, GNS 2009–10, Vietnamese General Nutrition Survey 2009–2010, NCDs, non-communicable diseases, NIN, National Institute of Nutrition, RAE, Retinol Activity Equivalent, sd, standard deviation, T, tertile, VHEI, Vietnamese Healthy Eating Index

## Abstract

Poor dietary quality is a major contributor to malnutrition and disease burden in Vietnam, necessitating the development of a tool for improving dietary quality. Food-based dietary guidelines (FBDGs) have been proposed to do this by providing specific, culturally appropriate and actionable recommendations. We developed the Vietnamese Healthy Eating Index (VHEI) to assess the adherence to the 2016–2020 Vietnamese FBDGs and the dietary quality of the general Vietnamese population. This VHEI consists of eight component scores, ‘grains’, ‘protein foods’, ‘vegetables’, ‘fruits’, ‘dairy’, ‘fats and oils’, ‘sugar and sweets’ and ‘salt and sauces’, representing the recommendations in the FBDGs. Each component score ranges from 0 to 10, resulting in a total VHEI score between 0 (lowest adherence) and 80 (highest adherence). The VHEI was calculated using dietary intake data from the Vietnamese General Nutrition Survey 2009–2010 (*n* = 8225 households). Associations of the VHEI with socio-demographic characteristics, energy and nutrient intakes and food group consumptions were examined. The results showed that the mean and standard deviation score of the VHEI was 43⋅3 ± 8⋅1. The component ‘sugar and sweets’ scored the highest (9⋅8 ± 1⋅1), whereas the component ‘dairy’ scored the lowest (0⋅6 ± 1⋅6). The intake of micronutrients was positively associated with the total VHEI, both before and after adjustment for energy intake. In conclusion, the VHEI is a valuable measure of dietary quality for the Vietnamese population regarding their adherence to the FBDGs.

## Introduction

Focusing on dietary patterns rather than on single foods or nutrients has been recommended in the literature as a more appropriate approach for exploring the relationships between diets and non-communicable diseases (NCDs)^(1,2)^. In recent years, scientific evidence on the relationships between diets and health outcomes has been translated into specific, culturally appropriate and actionable recommendations in the form of food-based dietary guidelines (FBDGs)^(3)^. These dietary guidelines are developed and regularly updated to influence the target population's nutritional behaviour and, in some countries, to inform a range of national food, nutrition and health policies and programmes^(3)^.

Association between adherence to the recommendations mentioned in the FBDGs and the related health outcomes should be examined to evaluate the potential impact of such FBDGs. For this purpose, a dietary quality index is needed to assess adherence to the FBDGs^(4–6)^. This type of index has been developed in many countries based on their current national FBDGs^(6–8)^ and has been used for various purposes, such as measuring the dietary quality of populations at one point in time or over a period of time^(9)^; assessing changes in dietary patterns in a nutritional intervention^(10)^; examining the associations between diets and diseases^(11)^ as well as the risk of mortality^(8)^; and combining environmental and other factors to assess the sustainability of foods and diets^(12)^.

Poor dietary quality is the main contributor to the burden of malnutrition and is one of the fundamental causes of morbidity and mortality worldwide, including Vietnam^(13,14)^. In Vietnam, NCDs such as cardiovascular diseases, cancers, chronic respiratory diseases and diabetes are main contributors to the disease burden, demonstrating the ongoing nutrition transition^(15,16)^. Also, the prevalence of overweight and obesity and hypertension in Vietnamese adults is increasing and in 2015 amounted to 15⋅0 % for overweight and 20⋅0 % for hypertension^(15)^. The traditional Vietnamese dietary pattern is considered to be low in fat, including small amounts of meat and fish, and rich in vegetables, high in salt and low in dairy^(17)^. However, the diets are quickly changing towards more unhealthy dietary patterns, with an increase in fat intake and meat consumption together with a decrease in vegetable intake^(16,17)^. This trend calls for national food policies and nutritional interventions, and the development of Vietnamese FBDGs with critical messages and visual representations as a tool for nutritional education and communication is among the key actions in achieving this goal^(18)^.

The Vietnamese FBDGs were first published in 1995 and have been revised every 5 years, aiming to promote healthy diets and serving as a basis for guidance on developing food and agriculture policies^(18,19)^. The 2016–2020 Vietnamese FBDGs are developed for different populations, including adults, pregnant and lactating women and children^(19,20)^. These current guidelines are developed based on the 2016 Vietnamese recommended dietary allowances^(21)^, the report ‘Ten tips on proper nutrition for the period 2011–2020’ in Vietnam^(19)^ and the results from studies on nutrition and health in the country, with adaptation from the international guidelines on nutrition and physical activity^(22)^. However, no index is currently in use that measures the adherence to these FBDGs to assess the dietary quality of Vietnamese adults. Thus, the present study aimed to develop the Vietnamese Healthy Eating Index (VHEI) as a measure of dietary quality in terms of adherence to the 2016–2020 Vietnamese FBDGs for adults and to examine the associations between the VHEI and socio-demographic characteristics, energy and nutrient intakes and food group consumptions of the study population.

## Subjects and methods

### Study population

The research described in this paper was based on an analysis of the Vietnamese General Nutrition Survey 2009–2010 (GNS 2009–2010). This survey aimed to determine the nutritional status and household food consumption of the Vietnamese population. The GNS 2009–2010 survey was conducted in accordance with the guidelines laid down in the Declaration of Helsinki and was approved by the Ethical Committee of the National Institute of Nutrition (NIN), Ministry of Health, Vietnam. Written informed consent was obtained from the participants prior to data collection^(23)^. NIN permitted full access to the dataset.

In the original GNS 2009–2010 survey, targeted households were selected by a stratified multi-stage cluster design across the six ecological zones in Vietnam. The sampling procedure of this survey has been described in more detail elsewhere^(23)^. The households included were those willing to participate and consisted of at least three members with one available adult responsible for food preparation, resulting in 8386 households.

### Dietary assessment

The food consumption data for the previous 24 h were collected by trained interviewers for each household. Briefly, food consumption was described by a representative household member who was responsible for preparing meals and beverages that all household members consumed. The edible portions, yield factors and conversion factors of the food items were applied^(23)^. To conduct the analysis and present results based on the intake of one person instead of the whole household, the Adult Male Equivalent (AME) concept was first introduced^(24)^. Although this approach does reflect individual intake, the AME represents a proxy for intra-household food distribution. It has been validated and used widely to convert household intake data to the intake of a reference individual based on energy requirements^(25,26)^. Studies have used the AME with Household Consumption and Expenditures Surveys data and found values were comparable to individual 24-h recall intake data^(24)^. However, since target groups in nutritional programmes are usually not the adult men but women of reproductive age, as they are among the most vulnerable groups and thus a highly relevant group of interest to include in research, the Adult Female Equivalent (AFE) referring to an adult non-pregnant non-lactating woman, 20–30 years, was used as suggested previously^(27)^. In the present study, we followed the AFE strategy to transform household food consumption into intake of a reference individual^(24)^, with a correction based on the recommendations of the Human Energy Requirements^(28)^ for all individuals in the households, considering their age and gender. AFE values of other household members were calculated by dividing their energy requirement by the energy requirement of the reference AFE per day and they were then summed up to obtain the total household AFE. Because information about age and sex was missing for members of 145 households, these households were deleted. Outliers were identified based on a *Z*-score value of less than −2⋅58 or more than 2⋅58 derived from energy intake, resulting in the deletion of 16 extreme outliers and leaving 8225 observations in our final analysis.

The 2019 Vietnamese Food Composition Table (FCT)^(29)^ was used as the primary source to estimate energy and nutrient intakes. Approximately 20 % of the energy and nutrient values of 613 food items in the GNS 2009–2010 were missing and complemented with data from the 2017 Indian FCT^(30)^, the 2015 Standard FCT in Japan^(31)^, the 2020 Food Data Central and The US Department of Agriculture^(32)^ (mentioned in order of use).

### Development of the VHEI

We created the VHEI to measure adherence to the 2016–2020 FBDGs for Vietnamese adults aged 20 years and older, with a higher score demonstrating higher adherence and thus higher dietary quality. The eight component scores were developed to reflect the recommendations relating to the eight food groups of the FBDGs, including ‘grains’, ‘protein foods’, ‘vegetables’, ‘fruits’, ‘dairy’, ‘fats and oils’, ‘sugar and sweets’ and ‘salt and sauce’. The information regarding the definition of servings of each food group and the foods to be included in each food group were derived from the graphic presentation (Supplementary Fig. S1), the official background document of the 2016–2020 Vietnamese FBDGs (written and published in Vietnamese)^(22)^ and information provided by the NIN, Ministry of Health, Vietnam. The graphic presentation is translated from Vietnamese into English and gathered with additional information from the official background document of the FBDGs in Supplementary Table S1. The eight components were divided into adequacy, moderation and optimum categories, with a different scoring system for each category as further described below.

#### Adequacy category

‘Vegetables’ and ‘fruits’ are classified as adequacy categories. These food groups are considered healthy; thus, participants earn higher scores if they consume more of them. We modified recommended serving for vegetables and fruits to remove the upper limit of intake (described clearly below) in conforming to other scoring systems^(8,33)^.

##### Vegetables

The component ‘vegetables’ was formulated based on the recommendation in the FBDGs that a Vietnamese adult should consume 3–4 servings of vegetables per day but was adapted to be 3 servings or more, with 80 g of raw edible vegetables constituting one serving. Food items for this component encompassed all types of vegetables, including frozen and canned vegetables, mushrooms and peas, but not legumes or potatoes. Vegetable juices were not included in this component due to the low fibre content. In the case of vegetable soup, vegetable broth was not classified as a vegetable, and we only counted the proportion of vegetables.

##### Fruits

The component ‘fruits’ was formulated based on the recommendation in the FBDGs that a Vietnamese adult should consume three servings of fruits per day but was adapted to be 3 servings or more, with 80 g of edible fruit constituting one serving. Food items for this component encompassed all types of fruits, including frozen fruit. However, dried fruit, canned fruit, fruit juices and fruit smoothies were not included in this component due to the high sugar content.

##### Scoring system for adequacy category

A minimum score of 0 was assigned when participants did not consume any items in this category. A maximum score of 10 was assigned when participants consumed equal to or more than the recommended servings. When participants consumed less than the recommended servings, the score was calculated with the following formula:
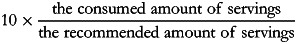


#### Optimum category

‘Grains’, ‘protein foods’, ‘fats and oils’ and ‘dairy’ are classified as optimum categories as the intake should be within an optimal range. Thus, participants score lower if their intake is above the upper limit or below the lower limit of the optimal range.

##### Grains

The component ‘grains’ was formulated based on the recommendation that 12–15 servings of grains should be consumed daily, with one serving of ‘grains’ containing 20 g carbohydrate. Examples of one serving described in the FBDGs are 55 g cooked rice, 37 g bread or 95 g potato. Food items included in this component are rice (plain rice, fried rice, broken rice, glutinous rice and porridge rice), bread (white bread or whole grain bread), noodles (rice-based noodles, wheat-based noodles and instant noodles), potato (white potato, sweet potato and Chinese yam) and maize. No distinction was made for whole grains in this component.

##### Protein foods

The component ‘protein foods’ was formulated based on the recommendation that 5–6 servings of protein foods should be consumed daily, with one serving of ‘protein foods’ containing 7 g protein. Examples of one serving described in the FBDGs are 31 g cooked pork, 42 g cooked chicken or 35 g cooked fish. Food items included in this component are all types of fresh, frozen or canned meat (whereby no distinction between red or white meat was made), fish, seafood, eggs, soyabean products and other legumes (excluding peas) but not dairy products.

##### Dairy

The component ‘dairy’ was formulated based on the recommendations that 3–4 servings of dairy and dairy products should be consumed daily, with one serving of ‘dairy’ containing 100 mg calcium. Examples of one serving described in the FBDGs are 100 ml milk, 100 g yogurt and 15 g cheese. Food items included in this component were milk, milk powder, yogurt and cheese, whereas sugar-sweetened dairy (condensed milk) and soya milk were not included.

##### Fats and oils

The component ‘fats and oils’ was formulated based on the recommendation that 5–6 servings of fats and oils should be consumed daily, with one serving of ‘fats and oils’ containing 5 g total fat. Food items included in this component were cooking oil, vegetable oil, animal fat, butter, margarine, nuts and seeds.

##### Scoring system for optimum category

A maximum score of 10 points was assigned if participants consumed within the optimal range. When participants consumed less than the lower limit of the optimal range, the score was calculated with the following formula:
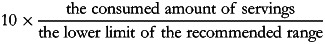


When participants consumed more than the upper limit of the optimal range, the score was calculated with the following formula:
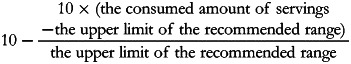


#### Moderation category

‘Sugar and sweets’ and ‘salt and sauces’ are classified as moderation categories. These food groups are considered unhealthy; thus, participants earn higher scores if they consume less of them.

##### Sugar and sweets

The component ‘sugar and sweets’ was formulated based on the recommendation that less than 5 servings of sugar and sweets should be consumed daily, with one serving of ‘sugar and sweets’ containing 5 g sugar. Examples of one serving described in the FBDGs are 5 g table sugar, 6 g honey and 8 g candy. Food items included in this component were added sugar, sugar-containing products such as candy, cakes, biscuits and desserts, sugar-sweetened dairy (condensed milk) and sugar-sweetened soft drinks. Instant drink powders (coffee, cocoa, orange flavour, etc.), dried or canned fruit, fruit juices and smoothies were also included due to their high sugar content.

##### Salt and sauces

The component ‘salt and sauces’ was formulated based on the recommendation that less than 1 serving of salt and sauces should be consumed daily, with one serving of ‘salt and sauces’ containing 5 g of table salt (equal to 1938 mg of sodium in seasonings and sauces). Examples of one serving described in the FBDGs are 8 g seasoning powder, 25 g fish sauce or 35 g soya sauce. Food items included in this component were table salt, salt-containing products such as seasoning powder, fish sauce, soya sauce and chilli sauce, added during cooking or at the table.

##### Scoring system for moderation category

**Fig. 1.**
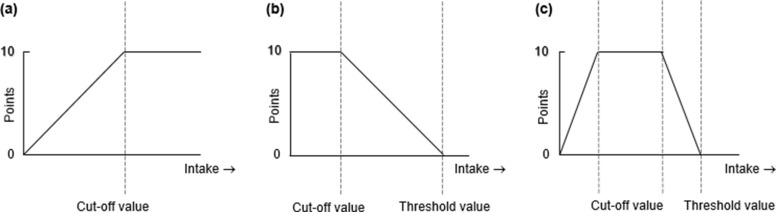
Graphical presentation of the Vietnamese Healthy Eating Index for (a) adequacy category, (b) moderation category and (c) optimum category

A maximum score of 10 points was assigned when participants consumed less than the recommended servings. When participants consumed more than the recommended servings, the score was calculated with the formula:
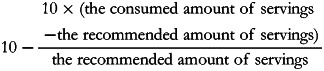


The scores of the eight components would be rounded off to the nearest whole number and capped at 0 if the calculations provided a negative score. They were then summed up to obtain a total VHEI score ranging from 0 (the lowest adherence to the Vietnamese FBDGs) to 80 (the highest adherence to the Vietnamese FBDGs). An overview of eight component scores and their cut-off values (maximum score) and threshold values (minimum score) are summarised in Table 1 and visually illustrated in Fig. 1, which is adapted from the study by Looman *et al.*^(34)^.

**Table 1.** Operationalisation of the Vietnamese Healthy Eating Index

Components of the VHEI*	Cut-off value (minimum score 0 points)	Scoring between 0 and 10 points	Threshold value (maximum score 10 points)
Adequacy category			
Vegetables	0	>0–<3	≥3
Fruits	0	>0–<3	≥3
Optimum category			
Grains	0 or ≥30	>0–<12 or >15–<30	12–15
Protein foods	0 or ≥12	>0–<5 or >6–<12	5–6
Fats and oils	0 or ≥12	>0–<5 or >6–<12	5–6
Dairy	0 or ≥8	>0–<3 or >4–<8	3–4
Moderation category			
Sugar and sweets	≥10	≥5–<10	<5
Salt and sauces	≥2	≥1–<2	<1

VHEI, Vietnamese Healthy Eating Index, with a range from 0 to 80 points.

* Data are presented in serving.

### Statistical analysis

Sample weights of primary sampling units in the survey design were applied as provided by NIN^(23)^. Data were presented as mean and standard deviation for continuous variables and as percentages of participants for categorical variables. Mean across tertiles of the VHEI score were compared using *P-value* for trend based on general linear models to examine the associations between the total VHEI score and the socio-demographic characteristics, energy intakes, nutrient intakes, and food group consumption. Concordance of ranking of participants based on their adherence using the VHEI was examined by calculating Spearman's rank correlation coefficients. Nutrient intake was reported with and without energy adjustment. Adjusted macronutrient intake was presented as energy percentage (*E* %), and adjusted micronutrient intake was presented as mean intake per 1000 kcal. All statistical analyses were performed using the statistical software package Stata version 15, and a *P*-*value* of <0⋅05 was considered statistically significant.

## Results

**Fig. 2.**
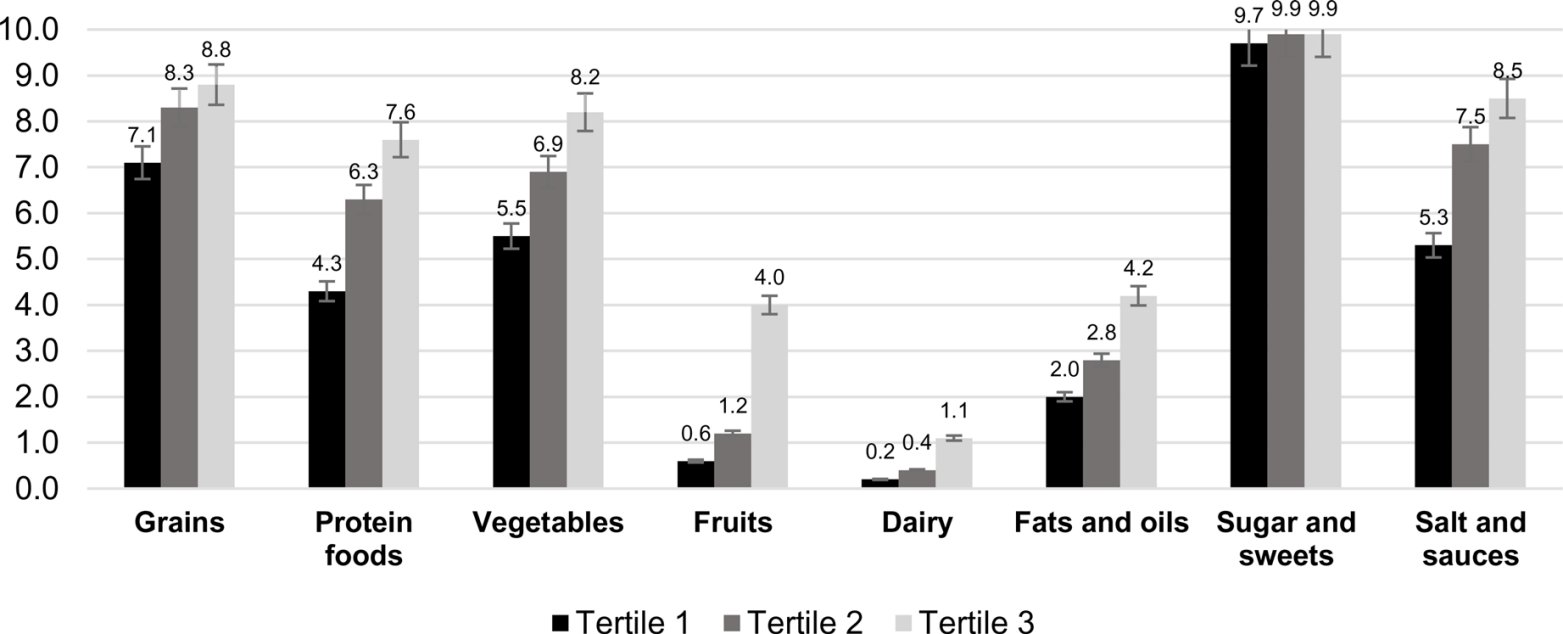
Mean component scores across tertiles of the Vietnamese Healthy Eating Index (Tertile 1 = lowest score = lowest adherence = lowest dietary quality), mean values adjusted using sample weights

The mean score of the VHEI was 43⋅3 ± 8⋅1 and ranged from 12⋅7 to 72⋅1 out of a possible total of 80. The highest mean score was observed for the component ‘sugar and sweets’ (9⋅8 ± 1⋅1) followed by the component ‘grains’ (8⋅1 ± 2⋅3), whereas the lowest mean score was found for the component ‘dairy’ (0⋅6 ± 1⋅6) followed by the component ‘fruits’ (1⋅8 ± 3⋅1). The mean score of the component ‘fats and oils’ was also low, with a mean of 3⋅1 ± 2⋅9. The scores of components ‘protein foods’, ‘vegetables’ and ‘salt and sauces’ were 6⋅0 ± 3⋅1, 6⋅9 ± 2⋅9 and 7⋅0 ± 3⋅8, respectively. The mean scores of each component across tertiles of the VHEI are presented in Fig. 2. All the component scores showed significant positive trends across tertiles of the VHEI as examined by the general linear model (*P-value* for trend <0⋅001).

There was a significant association between the VHEI and region, as shown in Table 2. In the highest tertile of the VHEI large part of the households were from the Red River delta (28⋅9 %), whereas in the lowest tertile of the VHEI more households were from the Northern and central coastal areas (33⋅2 %), followed by Mekong River delta (25⋅0 %). Approximately 50 % of the participants in the first tertile were from the two lowest income groups, while more than 50 % of the participants in the third tertile were from the two highest income groups.

**Table 2.** Socio-demographic characteristics of participants in the Vietnamese General Nutrition Survey 2009–2010 (*n* 8225) across tertiles of the Vietnamese Healthy Eating Index

Socio-demographic characteristics*	Tertiles of total VHEI score			*P*-*value*^†^
	T1 (*n* 2742)	T2 (*n* 2742)	T3 (*n* 2741)	
Regions (%)				<0⋅001
Red River delta	13⋅5	19⋅7	28⋅8	
Northern midlands and mountain areas	13⋅6	19⋅3	18⋅0	
Northern and central coastal areas	33⋅2	25⋅3	17⋅0	
Central highland	7⋅1	4⋅9	4⋅1	
Southeast	7⋅6	11⋅6	18⋅4	
Mekong River delta	25⋅0	19⋅2	13⋅7	
Household income (%)				<0⋅001
Lowest	22⋅9	15⋅0	10⋅0	
Second	24⋅8	21⋅0	11⋅5	
Third	19⋅4	22⋅8	19⋅4	
Fourth	19⋅9	23⋅2	23⋅4	
Highest	13⋅0	18⋅0	35⋅7	

T, tertile (T1 = lowest score = lowest adherence = lowest dietary quality); VHEI, Vietnamese Healthy Eating Index, with a range from 0 to 80 points.

* Data are presented in percentage (%).

† *P*-*value* for trend analysed by general linear model.

Over the tertiles, no clear difference in energy intake was observed, as presented in Table 3. The VHEI was positively associated with the intake of protein, dietary fibre, total fat, and monounsaturated, polyunsaturated and saturated fatty acids. These positive associations remained unchanged after adjustment for energy intake. For carbohydrate intake, an inverse trend across tertiles of the VHEI was observed both before and after adjusting for energy intake. There were moderate positive correlations between the VHEI and intake of protein, dietary fibre, total fat and fatty acids, both before and after energy adjustment (*r_s_* ranged from 0⋅23 to 0⋅36). There was a negative correlation between the VHEI and carbohydrate intake, with a poor correlation before energy adjustment and a moderate inverse correlation after energy adjustment (*r_s_* −0⋅08 and *r_s_* −0⋅35, respectively). The correlation between the VHEI and energy intake was weak, with *r_s_* 0⋅06.

**Table 3.** Daily macronutrient intake of participants in the Vietnamese General Nutrition Survey 2009–2010 (*n* 8225) across tertiles of the Vietnamese Healthy Eating Index

Macronutrients* (AFE/d)	Tertiles of total VHEI score						*P*-*value*^†^	*r_s_*
	T1 (*n* 2742)		T2 (*n* 2742)		T3 (*n* 2741)			
Energy intake (kcal)	1917	860⋅3	1814	563⋅4	1939	454⋅0	0⋅958	0⋅06^‡^
Macronutrient intake (g)								
Protein	67⋅1	36⋅6	68⋅8	26⋅9	74⋅9	20⋅2	<0⋅001	0⋅23^‡^
Carbohydrate	339⋅3	155⋅4	305⋅7	100⋅9	310⋅6	76⋅5	0⋅001	−0⋅08^‡^
Dietary fibre	5⋅2	3⋅9	5⋅6	3⋅8	7⋅3	3⋅8	<0⋅001	0⋅28^‡^
Total fat	30⋅5	32⋅8	33⋅2	23⋅7	41⋅7	23⋅4	<0⋅001	0⋅32^‡^
Saturated fat	9⋅4	13⋅1	10⋅0	8⋅1	12⋅3	8⋅2	0⋅001	0⋅29^‡^
Monounsaturated fat	12⋅7	15⋅2	13⋅9	10⋅7	17⋅7	11⋅0	<0⋅001	0⋅33^‡^
Polyunsaturated fat	6⋅7	8⋅7	7⋅2	6⋅1	9⋅5	5⋅9	<0⋅001	0⋅36^‡^
Energy percentage macronutrient intake (E%)								
Protein	14⋅1	4⋅5	15⋅2	3⋅6	15⋅6	2⋅8	<0⋅001	0⋅28^‡^
Carbohydrate	71⋅4	12⋅8	67⋅7	10⋅0	64⋅4	8⋅1	<0⋅001	−0⋅35^‡^
Total fat	13⋅6	10⋅2	16⋅0	8⋅4	18⋅9	7⋅5	<0⋅001	0⋅33^‡^
Saturated fat	4⋅2	3⋅9	4⋅9	3⋅2	5⋅6	3⋅2	<0⋅001	0⋅27^‡^
Monounsaturated fat	5⋅7	5⋅3	6⋅7	4⋅3	8⋅1	4⋅4	<0⋅001	0⋅32^‡^
Polyunsaturated fat	3⋅0	3⋅0	3⋅5	2⋅5	4⋅4	2⋅5	<0⋅001	0⋅36^‡^

AFE, Adult Female Equivalent; T, Tertile (T1 = lowest score = lowest adherence = lowest dietary quality); VHEI, Vietnamese Healthy Eating Index, with a range from 0 to 80 points.

* Data are presented in mean and standard deviation, mean adjusted for sample weights.

† *P*-*value* for trend analysed by general linear model.

‡ A statistical significance with a *P*-*value* of <0⋅001 calculated from Spearman's rank correlation.

Intake of calcium, potassium, magnesium, iron, zinc, folate, thiamine, riboflavin, vitamin C, vitamin A Retinol Activity Equivalent (RAE), vitamin B6 and vitamin B12 were positively associated with the VHEI, both before and after energy intake adjustment. The significant correlations between the VHEI and the micronutrient intake was confirmed by the Spearman's rank correlation coefficients (*P-value* < 0⋅001). For each of the essential micronutrients (calcium, potassium, magnesium, iron, zinc, folate, riboflavin, thiamine, vitamin A RAE, vitamin C, vitamin B6 and vitamin B12), there was a positive correlation between the VHEI and the intake of these nutrients, both before and after energy adjustment (with *r_s_* ranging from 0⋅16 to 0⋅36). There was a negative correlation between the VHEI and sodium intake, both before and after energy adjustment (*r_s_* −0⋅16 and *r_s_* −0⋅20, respectively), as shown in Table 4.

**Table 4.** Daily micronutrient intake of participants in the Vietnamese General Nutrition Survey 2009–2010 (*n* 8225) across tertiles of the Vietnamese Healthy Eating Index

Micronutrients* (AFE/d)	Tertiles of total VHEI score						*P*-*value*^†^	*r_s_*
	T1 (*n* 2742)		T2 (*n* 2742)		T3 (*n* 2741)			
Micronutrient intake								
Calcium (mg)	388⋅3	297⋅8	405⋅1	250⋅6	488⋅9	323⋅6	<0⋅001	0⋅24^‡^
Potassium (mg)	1938	988⋅3	1998	732⋅1	2362	660⋅4	<0⋅001	0⋅27^‡^
Sodium (mg)	3600	3240	2870	2409	2521	1564	<0⋅001	−0⋅16^‡^
Magnesium (mg)	200⋅3	139⋅1	204⋅7	126⋅5	233⋅1	96⋅9	<0⋅001	0⋅23^‡^
Iron (mg)	11⋅6	11⋅0	12⋅2	12⋅9	13⋅9	8⋅5	0⋅029	0⋅23^‡^
Zinc (mg)	9⋅8	4⋅9	9⋅8	3⋅8	10⋅9	3⋅7	0⋅002	0⋅16^‡^
Folate (μg)	186⋅7	151⋅7	221⋅1	150⋅3	274⋅4	150⋅6	<0⋅001	0⋅28^‡^
Riboflavin (mg)	0⋅6	0⋅4	0⋅7	0⋅4	0⋅8	0⋅5	<0⋅001	0⋅31^‡^
Thiamine (mg)	0⋅8	0⋅5	0⋅8	0⋅4	1⋅0	0⋅4	<0⋅001	0⋅26^‡^
Vitamin A RAE (μg)	356⋅4	438⋅6	435⋅1	426⋅7	549⋅0	515⋅5	<0⋅001	0⋅22^‡^
Vitamin C (mg)	43⋅1	52⋅2	52⋅5	42⋅9	85⋅8	68⋅8	<0⋅001	0⋅36^‡^
Vitamin B6 (μg)	1⋅3	0⋅7	1⋅3	0⋅6	1⋅5	0⋅6	<0⋅001	0⋅25^‡^
Vitamin B12 (μg)	2⋅1	3⋅5	2⋅4	3⋅5	2⋅9	5⋅5	<0⋅001	0⋅25^‡^
Energy-adjusted micronutrient intake								
Calcium (mg/1000 kcal)	212⋅1	156⋅3	228⋅7	134⋅1	255⋅7	153⋅9	<0⋅001	0⋅24^‡^
Potassium (mg/1000 kcal)	1038	355⋅6	1118	283⋅0	1236	276⋅3	<0⋅001	0⋅32^‡^
Sodium (mg/1000 kcal)	2016	1737	1611	1254	1325	770⋅1	<0⋅001	−0⋅20^‡^
Magnesium (mg/1000 kcal)	107⋅1	57⋅1	113⋅8	56⋅9	121⋅7	43⋅5	<0⋅001	0⋅22^‡^
Iron (mg/1000 kcal)	6⋅1	3⋅3	6⋅7	4⋅1	7⋅2	3⋅2	<0⋅001	0⋅27^‡^
Zinc (mg/1000 kcal)	5⋅2	1⋅2	5⋅4	1⋅1	5⋅6	1⋅4	<0⋅001	0⋅20^‡^
Folate (μg/1000 kcal)	102⋅8	92⋅1	125⋅2	83⋅6	144⋅2	76⋅5	<0⋅001	0⋅27^‡^
Riboflavin (mg/1000 kcal)	0⋅3	0⋅2	0⋅4	0⋅2	0⋅4	0⋅2	<0⋅001	0⋅32^‡^
Thiamine (mg/1000 kcal)	0⋅4	0⋅2	0⋅5	0⋅2	0⋅5	0⋅2	<0⋅001	0⋅29^‡^
Vitamin A RAE (μg/1000 kcal)	197⋅8	264⋅2	245⋅0	243⋅3	287⋅2	256⋅0	<0⋅001	0⋅21^‡^
Vitamin C (mg/1000 kcal)	24⋅3	29⋅9	30⋅1	25⋅7	45⋅2	35⋅5	<0⋅001	0⋅34^‡^
Vitamin B6 (μg/1000 kcal)	0⋅7	0⋅2	0⋅7	0⋅2	0⋅8	0⋅2	<0⋅001	0⋅29^‡^
Vitamin B12 (μg/1000 kcal)	1⋅1	1⋅8	1⋅3	1⋅9	1⋅5	3⋅0	0⋅001	0⋅22^‡^

AFE, Adult Female Equivalent; T, tertile (T1 = lowest score = lowest adherence = lowest dietary quality); VHEI, Vietnamese Healthy Eating Index, with a range from 0 to 80 points.

* Data are presented in mean values and standard deviation, mean values adjusted for sample weights.

† *P*-*value* for trend analysed by general linear model.

‡ A statistical significance with a *P*-*value* of <0⋅001 calculated from Spearman's rank correlation.

Rice was the main food item contributing to the intake of grains food group (Table 5). There was an inverse association between rice consumption and the VHEI, whereas the consumption of noodles was positively associated with the VHEI (Table 5). Within the protein foods group, no association between the VHEI and the consumption of white meat, fish and seafood was observed. Positive associations were observed between the VHEI and the other protein foods such as red meat, eggs, soyabean and legumes. Significant positive associations across tertiles of the VHEI were seen for the consumption of the food groups dairy and dairy products, vegetables, fruits, and fats and oils. A significant inverse association was observed for the food group salt and sauces. Low inverse correlations between the VHEI and grains, rice, and salt and sauces consumptions were observed (*r_s_* −0⋅10, −0⋅14 and −0⋅10, respectively). Correlation coefficients between the VHEI and other food groups consumption were positive, ranging from 0⋅05 to 0⋅38.

**Table 5.** Daily food group consumption of participants in the Vietnamese General Nutrition Survey 2009–2010 (*n* 8225) across tertiles of the Vietnamese Healthy Eating Index

Food groups* (g/AFE/d)	Tertiles of total VHEI score						*P*-*value*^†^	*r_s_*
	T1 (*n* 2742)		T2 (*n* 2742)		T3 (*n* 2741)			
Grains	434⋅0	209⋅1	388⋅7	134⋅0	383⋅2	101⋅3	<0⋅001	−0⋅10^‡^
Rice	392⋅8	201⋅2	346⋅6	131⋅4	330⋅0	104⋅1	<0⋅001	−0⋅14^‡^
Noodles	28⋅0	70⋅1	34⋅1	63⋅3	44⋅3	67⋅7	<0⋅001	0⋅15^‡^
Bread	3⋅6	21⋅5	3⋅1	15⋅6	4⋅8	17⋅0	0⋅053	0⋅05^‡^
Protein foods	159⋅2	167⋅5	186⋅8	128⋅4	218⋅8	100⋅6	<0⋅001	0⋅32^‡^
White meat	13⋅2	58⋅3	14⋅2	66⋅8	14⋅6	45⋅9	0⋅882	0⋅05^‡^
Red meat	54⋅3	91⋅7	66⋅1	82⋅1	84⋅9	75⋅8	<0⋅001	0⋅22^‡^
Fish and seafood	64⋅4	99⋅5	71⋅0	86⋅3	68⋅5	73⋅0	0⋅084	0⋅09^‡^
Eggs	9⋅1	24⋅3	13⋅1	26⋅5	15⋅8	25⋅4	<0⋅001	0⋅15^‡^
Soyabean and legumes	17⋅3	76⋅5	21⋅1	54⋅4	33⋅4	68⋅8	0⋅001	0⋅16^‡^
Dairy and dairy products	4⋅3	22⋅6	8⋅3	30⋅7	19⋅1	44⋅8	<0⋅001	0⋅22^‡^
Vegetables	153⋅9	131⋅1	191⋅2	115⋅7	237⋅5	110⋅9	<0⋅001	0⋅32^‡^
Fruits	17⋅3	64⋅7	33⋅0	79⋅3	119⋅1	139⋅3	<0⋅001	0⋅38^‡^
Sugar and sweets	7⋅7	24⋅7	6⋅1	32⋅0	5⋅7	14⋅4	0⋅133	0⋅02^‡^
Fats and oils	11⋅6	38⋅5	11⋅1	21⋅8	14⋅6	17⋅5	0⋅031	0⋅30^‡^
Salt and sauces	22⋅6	21⋅7	16⋅9	19⋅0	15⋅1	12⋅7	<0⋅001	−0⋅10^‡^

AFE, Adult Female Equivalent; T, tertile (T1 = lowest score = lowest adherence = lowest dietary quality); VHEI, Vietnamese Healthy Eating Index, with a range from 0 to 80 points.

* Data are presented in mean values and standard deviation, mean values adjusted for sample weights.

† *P*-*value* for trend analysed by general linear model.

‡ A statistical significance with a *P*-*value* of <0⋅001 calculated from Spearman's rank correlation.

## Discussion

We developed the VHEI as a measure of dietary quality in terms of adherence to the 2016–2020 FBDGs Vietnamese for adults. The index proved to be a valuable tool for ranking participants based on their adherence to the FBDGs in our analysis. A positive association was observed between the VHEI and energy-adjusted intake of micronutrients calcium, potassium, magnesium, iron, zinc, folate, thiamine, riboflavin, vitamin A RAE, vitamin C, vitamin B6 and vitamin B12, suggesting that a higher VHEI score was associated with higher dietary quality. The total VHEI score was also positively associated with protein, total fat and dietary fibre intake and inversely associated with sodium and sugar intake. There was a positive relationship between the total VHEI score and region and households’ income.

The eight component scores of the VHEI ‘grains’, ‘protein foods’, ‘vegetables’, ‘fruits’, ‘dairy’, ‘fats and oils’, ‘sugar and sweets’ and ‘salt and sauces’ were developed based on the recommendations for the eight food groups in the 2016–2020 Vietnamese FBDGs. Each component was scored individually based on the absolute amount consumed estimated in servings, instead of using consumption as a dichotomous variable (i.e., ‘yes’ or ‘no’). This is advantageous as it enables the possibility of grading within the score, however also it requires data about actual amounts consumed, which is challenging in low resource settings such as Vietnam^(5)^. Despite this short-coming, the flexibility of a graded system is still superior to a binary system that sheds no light on quantities of the foods consumed.

We gave a similar weight to the eight component scores in order to reflect the equal weighting seen in the Vietnamese FBDGs. This approach is applied widely in the literature^(7,8,34)^ and is generally suggested when developing a dietary quality index^(5)^. However, different components of a healthy eating index may affect the total dietary quality score differently^(35)^. For example, the Chinese Healthy Eating Index applied unequal weighting factors for different individual components, in that cooking oils, sodium and fruits were weighted twice as heavily as other components. They were considered important in Chinese dietary patterns and linked with various adverse health outcomes^(35)^. Future research may explore whether applying weighting factors for different components could improve the efficacy of a dietary quality index in Vietnam.

The FBDGs also give recommendations on water consumption and physical activity; however, these were not included in our methodology as the dietary intake dataset did not cover water consumption or physical activity information. Other indices did capture physical activity^(7,8,36)^, while others did not^(7,34,37)^. Water intake and physical activity are integral parts of a healthy diet and lifestyle and their incorporation could be expected to increase the efficacy of a dietary quality index. Thus, it is advisable that these components are also included when collecting data or modifying the VHEI.

Participants who had a higher adherence to the Vietnamese FBDGs had higher absolute intake and higher energy-adjusted intake of micronutrients. This outcome demonstrates that a higher intake of micronutrients in the diet was observed among those who adhered more closely to the FBDGs regardless of their energy intake, which is in line with other studies^(7,34,37)^. The mean intake of micronutrients vitamin B2 and calcium was considerably lower than the recommended average intake for Vietnamese adults in all tertiles of the VHEI^(21)^. In Vietnam, calcium deficiency is still a severe problem affecting people of all ages and consumption of dairy and dairy products is still low^(38,39)^, as shown in our study where ‘dairy’ scored the lowest among the eight component scores. The low intake of dairy and dairy products elsewhere in Asia was explained by the low per-capita supply and widespread lactose intolerance and lactase deficiency^(35)^, though evidence is currently absent in Vietnam, preventing definitive conclusions from being made.

A higher total fat intake was observed for participants in the highest tertile of the VHEI, which differs from other studies that found higher dietary quality scores associated with lower total fat intake^(34,35)^. The low consumption of total fat in our study population could partly explain this result. The energy percentage of total fat intake ranged from 13⋅6 % in the lowest tertile to 18⋅9 % for participants in the highest tertile, which were lower than the recommended value for Vietnamese adults (20–25 %)^(21)^. Similarly, the absolute intake was low, ranging from 11⋅6 to 14⋅6 g, compared to the value of 25–30 g as recommended in the FBDGs. As a result, those scoring higher on ‘fats and oils’ component typically had values closer to the recommended value. In our study, saturated fat intake increased across tertiles of the VHEI, although it could be expected to decrease as unsaturated fat is preferable to saturated fat from a health perspective^(40)^. However, in the current Vietnamese FBDGs, no distinction is made between healthy and unhealthy fats, which are captured together in one ‘fats and oils’ group, which is similar to that which is seen in the FBDGs of 35 % of other countries^(3)^. Since we based our index on existing recommendations in the FBDGs, we also utilised only ‘fats and oils’ component, although other dietary quality indices separate total fat and saturated fat components^(34,41,42)^. New versions of the Vietnamese FBDGs should distinguish between different types of fat, as well as having a category for total overall fat in order to better reflect dietary quality.

Although most participants were assigned a relatively high score for the ‘grains’ component, almost none of them consumed whole grain products, indicating that the most significant proportion of the total grains consumed consisted of refined grains such as white rice, white noodle and white bread. This is undesirable given evidence for the negative effects of refined grains on health, such as their association with an increased risk of type 2 diabetes^(43)^. Whole grains also contain a considerable amount of fibre, which partially explains our participants’ low dietary fibre intake. These health advantages of whole grain warrant its preference over refined grain in a healthy diet^(44,45)^. In other FBDGs, a clear recommendation of whole grain intake was made that 90 g of whole grain products should be consumed daily and they should replace refined grain products^(34)^. Thus, the Vietnamese FBDGs should consider updating the recommendation on grains to bias the intake of whole grains over refined grains.

Low intake of fruit and vegetables has been observed in another study in Vietnam, confirming that approximately 80 % Vietnamese adults consume less than five servings of fruits and vegetables daily^(46)^. Our study found similar results, as reflected by the lower scores for the ‘fruits’ component. The mean daily intake of fruits of participants in the first tertile and the second tertile was very low, with 17⋅3 and 33⋅0 g, respectively. Although participants in the third tertile had meaningfully higher fruits intake with the mean of 119⋅1 g/d, this number only met approximately 50 % of the recommendation that at least three servings (240 g/d) should be consumed.

The mean daily consumption of vegetables of participants in the highest tertile (237⋅5 g/d) was close to the recommendation of at least three servings (240 g/d), whereas the mean intakes of participants in the other two tertiles were lower (153⋅9 g/d in the first tertile and 191⋅2 g in the second tertile). Despite this existing failure to meet vegetable intake recommendation, it is also possible that consumption of vegetables was overestimated in our study due to missing information on the proportions of water and vegetables in vegetable soups. We have tried to correct this by using the information given in standard recipes, but this strategy may not have fully recovered the vegetables intake. Thus, there is an obvious necessity to amend this low vegetable intake that is apparent in the Vietnamese population in order to improve dietary quality.

The study participants scored reasonably highly for the component ‘salt and sauces’ due to the low intake of salt and sauces, despite other studies showing that the dietary salt intake in Vietnam is higher than the recommended value^(47)^. This discrepancy could be explained by the lack of clear information on salt or sauces added at the table or during cooking in our study. The majority (approximately 80 %) of salt intake comes from table salt or salty condiments at home, and this is especially true in the Vietnamese situation^(47)^. To resolve these differences, dietary intake studies in the future should estimate sodium intake more precisely and consider measuring sodium via 24 h collected urine samples, as this is the golden standard in research quantifying sodium intake^(48)^.

Data on individual characteristics were missing in our analysis since the dietary intake data were derived from a household food consumption survey. Thus, we could only examine a relationship between characteristics at the household level and the VHEI. Here, we found a positive association between the VHEI and household wealth, which is in accordance with the results of another study in Vietnam^(49)^. The VHEI also varied by region as the largest percentage of participants with higher VHEI scores were from the Red River delta. Another study also showed regional variation in dietary quality, where micronutrient (calcium and vitamin A) intake was higher in the Southeast and the Red River delta, and that the Red River delta had a more balanced dietary pattern than other regions in terms of macronutrient intake^(50)^. In contrast, participants with lower VHEI scores were more often from the Northern and central coastal areas and the Mekong River delta. Kim *et al.*^(50)^ also showed that inhabitants of the Mekong River delta had an excess energy intake from carbohydrates and a deficit of energy intake from other macronutrients. Regional differences also exist in terms of income, and these differences align with the aforementioned findings on dietary index scores. The Southeast, which includes the largest city Ho Chi Minh, and the Red River delta, which consists of the capital Hanoi, have the highest average incomes in Vietnam^(51)^. Thus, taking the results of the present study and findings of others into account^(49,50)^, it is rational to further explore if households with higher income have access to healthier foods that may be unaffordable to households with lower income, which positively impacts their dietary quality.

This present study has some limitations. First, the data we used were on the household level and needed to be converted to the individual level. This conversion might cause inaccuracy in estimating dietary intake since approximations were based on one reference household member's description of food consumption and the distribution among household members was not taken into account. This conversion had also prevented us from examining the VHEI and individual characteristics. Second, further work is needed to evaluate the VHEI regarding its reliability and validity. However, the VHEI is the first of its kind for Vietnam and was developed based on dietary intake data of a large national representative sample. This work was conducted in a low- and middle-income country, where tools and metrics are still lacking to fill in the knowledge gap of the relation between dietary quality and other aspects.

In conclusion, the VHEI proved it to be effective at measuring dietary quality in terms of adherence to the Vietnamese FBDGs, confirming it as a valuable tool for future research to examine the associations between dietary quality and health-related outcomes; relationships between dietary quality and the environmental impacts of diets; and affordability of diets in Vietnam. Additionally, the index can also be used as a monitoring tool in nutrition interventions focusing on improving dietary quality. Finally, and most importantly, the outcomes of our study provide recommendations for the improvement of the development of the new 2021–2025 Vietnamese FBDGs.

## Supplementary material

For supplementary material accompanying this paper visit https://doi.org/10.1017/jns.2022.44. click here to view supplementary material
