# Supplementary material for: Development of the Vietnamese Healthy Eating Index
Source: J Nutr Sci. 2022 Jun 9;11:e45. doi: 10.1017/jns.2022.44 (PMC9201877; doi:10.1017/jns.2022.44)
Supplement: Supplementary file 1 [file S2048679022000441sup001.pdf]

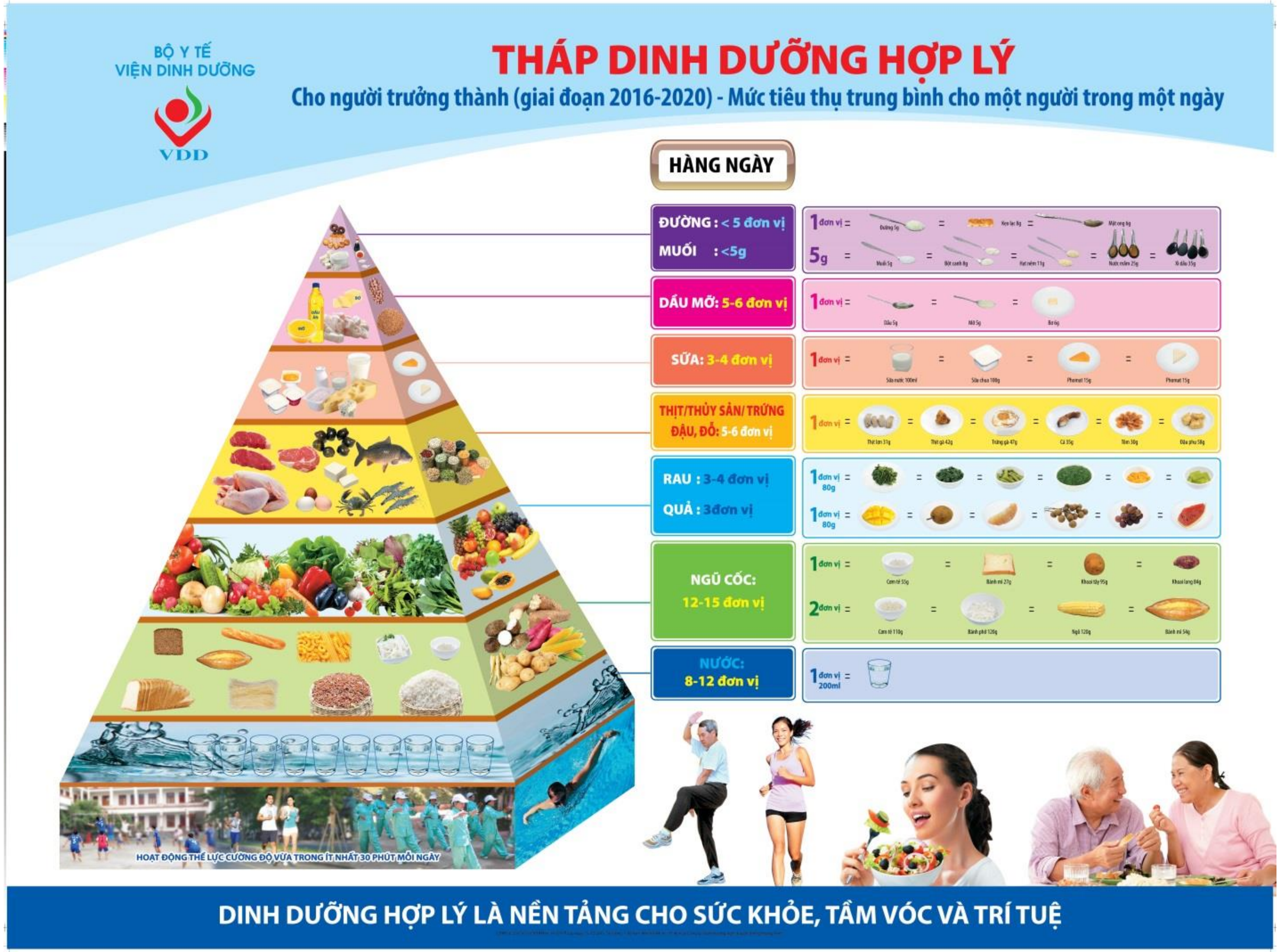

Figure S1. Visualization of the 2016-2020 Vietnamese food-based dietary guidelines for adults

Table S1. Interpretation of the 2016-2020 Vietnamese food-based dietary guidelines for adults*

Food groups	Main food items	Definition of ***one serving***	Examples of ***servings*** in the 2016-2020 FBDGs Pyramid	Recommended servings daily
**Grains**	Rice, bread, noodles, potato, corn, casava	Provides 20g carbohydrate	1 serving: 55g cooked rice, 27g sliced bread, 95g potato, 84g sweet potato<br>2 servings: 110g cooked rice, 120g cooked rice noodles, 120g cooked corn, 35g bread	12 – 15
**Protein foods**	Fish, meat, seafood, tofu and soybean products, egg	Provides 7g protein	1 serving: 31g cooked pork, 42g cooked chicken, 47g cooked egg, 35g cooked fish, 30g cooked shrimp, 58g cooked tofu	5 – 6
**Fats and oils**	Animal fats, oil, butter, nuts, and seeds	Provides 5g total fat	1 serving: 5g oils, 5g animal fats, 6g butter, 8g peanuts, 8g sesame seeds	5 – 6
**Dairy**	Milk, yogurt, cheese	Provides 100mg calcium	1 serving: 100ml milk, 100g yogurt, 15g cheese	3 – 4
**Vegetables**	All type of vegetables, mushroom	80g of edible vegetables (raw, cooked)	1 serving: 80g of water spinach, mustard green, Chinese cabbage, pumpkin, gourd	3 – 4
**Fruits**	All type of fruits	80g of edible fruits	1 serving: 80g of mango, lychee, grapefruits, longan, grape, watermelon	3
**Sugar and sweets**	Sugar, sweets, honey	Provides 5g free sugar	1 serving: 5g sugar, 8g peanut candy, 6g honey	< 5
**Salt and sauces**	Salt, seasoning, sauce	Provides 5g salt	1 serving: 5g salt, 8g seasoning powder, 25g fish sauce, 35g soy sauce	< 1
**Water**	Water	200ml plain water		8 – 12
**Physical activity**	At least 30 minutes of moderate physical activity every day.			

* The information in this table is the translation of the 2016-2020 food-based dietary guidelines pyramid with additional information derived from the 2016-2020 food-based dietary guidelines background document.
